# Oral Cannabidiol Use in Children With Autism Spectrum Disorder to Treat Related Symptoms and Co-morbidities

**DOI:** 10.3389/fphar.2018.01521

**Published:** 2019-01-09

**Authors:** Dana Barchel, Orit Stolar, Tal De-Haan, Tomer Ziv-Baran, Naama Saban, Danny Or Fuchs, Gideon Koren, Matitiahu Berkovitch

**Affiliations:** ^1^Clinical Pharmacology and Toxicology Unit, Assaf Harofeh Medical Center, Tel Aviv, Israel; ^2^Autistic Spectrum Disorder Clinic, Assaf Harofeh Medical Center, Tel Aviv, Israel; ^3^Department of Epidemiology and Preventive Medicine, School of Public Health, Sackler Faculty of Medicine, Tel Aviv University, Tel Aviv, Israel; ^4^Tikun Olam, Tel Aviv, Israel; ^5^Maccabi Institute for Health Services Research, Tel Aviv, Israel

**Keywords:** cannabidiol, autism spectrum disorder, ASD comorbid symptoms, ASD treatment, pediatrics, clinical research trial, THC – tetrahydrocannabinol

## Abstract

**Objective:** Children with autism spectrum disorder (ASD) commonly exhibit comorbid symptoms such as aggression, hyperactivity and anxiety. Several studies are being conducted worldwide on cannabidiol use in ASD; however, these studies are still ongoing, and data on the effects of its use is very limited. In this study we aimed to report the experience of parents who administer, under supervision, oral cannabinoids to their children with ASD.

**Methods:** After obtaining a license from the Israeli Ministry of Health, parents of children with ASD were instructed by a nurse practitioner how to administer oral drops of cannabidiol oil. Information on comorbid symptoms and safety was prospectively recorded biweekly during follow-up interviews. An independent group of specialists analyzed these data for changes in ASD symptoms and drug safety.

**Results:** 53 children at a median age of 11 (4–22) year received cannabidiol for a median duration of 66 days (30–588). Self-injury and rage attacks (*n* = 34) improved in 67.6% and worsened in 8.8%. Hyperactivity symptoms (*n* = 38) improved in 68.4%, did not change in 28.9% and worsened in 2.6%. Sleep problems (*n* = 21) improved in 71.4% and worsened in 4.7%. Anxiety (*n* = 17) improved in 47.1% and worsened in 23.5%. Adverse effects, mostly somnolence and change in appetite were mild.

**Conclusion:** Parents’ reports suggest that cannabidiol may improve ASD comorbidity symptoms; however, the long-term effects should be evaluated in large scale studies.

## Introduction

Children with autism spectrum disorder (ASD) commonly exhibit co-morbid symptoms of hyperactivity, self-injury, aggressiveness, restlessness, anxiety and sleep disorders (Mannion and Leader, 2013; South et al., 2017). Conventional medical treatment includes various psychotropic medications such as atypical anti psychotics, selective serotonin reuptake inhibitors (SSRI’s), stimulants and anxiolytics (Canitano and Scandurra, 2008; Stachnik and Gabay, 2010; Wink et al., 2010; Hurwitz et al., 2012).

Several studies are being conducted worldwide on the use of cannabidiol in children with ASD to treat comorbid symptoms. However, there is limited published data on the use of cannabinoids in this population (Kurz and Blaas, 2010; Kuester et al., 2017). A recent review has suggested cannabidiol as a candidate for treatment of ASD (Poleg et al., 2019). Cannabis contains numerous chemically active compounds, including Δ9-tetrahydrocannabinol (Δ9-THC), cannabidiol (CBD) and terpenoids (Russo, 2011). Δ9-THC activates the endocannabinoid system in the central nervous system, affecting appetite, anxiety, cognitive function and memory (Palmieri et al., 2017). In contrast, CBD is anxiolytic, anti-inflammatory, antiemetic and antipsychotic (Detyniecki and Hirsch, 2015). Studies in mice models of ASD have demonstrated the involvement of the endocannabinoid system in the pathogenesis of ASD symptoms (Foldy et al., 2013; Wei et al., 2015).

In this study we aimed to record the experience of parents who administered under supervision cannabidiol to their children with ASD.

## Materials and Methods

Included were children from all over Israel diagnosed with ASD based on DSM IV (American Psychiatric Association, 2000) or DSM V (American Psychiatric Association, 2013) criteria, between three and 25 years of age, who were followed up for at least 30 days after commencement of cannabidiol treatment. An independent group of specialists including a pediatric neurologist specialized in ASD, clinical pharmacologists and pharmacists objectively analyzed the data recorded during the follow up to assess symptom response and adverse effects. Four ASD comorbidity symptoms were evaluated: (a) hyperactivity symptoms (b) sleep problems, (c) self-injury and (d) anxiety.

For each comorbid symptom, the evaluations marked improvement, no change, or worsening of symptoms, as compared to the baseline, according to the parent’s reports. An overall change was defined based on the summation of all parent’s reports.

Children were recruited from a registry of patients with authorization to obtain cannabidiol (Tikun Olam Inc., Israel). Parents received a license for pediatric use of CBD from the Israeli Ministry of Health. The cannabinoid oil solution was prepared by “Tikun Olam” company, which is an approved supplier, at a concentration of 30% and 1:20 ratio of cannabidiol (CBD) and Δ9-tetrahydrocannabinol (THC). Quality assurance of the cannabidiol concentrations are routinely performed by HPLC on an Ultima 3000 Thermo Dionex instrument. Recommended daily dose of CBD was 16 mg/kg (maximal daily dose 600 mg), and for THC- daily dose of 0.8 mg/kg (maximal daily dose of 40 mg).

For all participating children this was their first experience with cannabidiol and no other cannabinoids were used before this study. During the first meeting, parents were instructed by an experienced nurse practitioner how to administer the preparation. Thereafter, a biweekly follow-up telephone interview was conducted with the parents. During the telephone interview, parents were asked on the status of the various ASD comorbid symptoms (graded as improvement, no change, worsening), emerging adverse effects and medications that had been used. Adverse events were coded using the Medical Dictionary for Regulatory Activities (Food Drug Administration, 2004). The change in each comorbid symptom in the study cohort was compared to published data using conventional treatment. For this purpose we used the following values: Hyperactivity symptoms- Improvement was considered as 80% (Handen et al., 2000), for self-injury an improvement was considered as 82% (Richards et al., 2016), for sleep problems an improvement was considered as 60% (Devnani and Hegde, 2015), and improvement in anxiety symptoms was considered as 64% (Moore et al., 2004).

### The Study Was Not Financially Supported by Tikun Olam Company

The study was approved by the local research ethics committee. The need for written parental consent for this study was waived by the Assaf Harofeh Medical Center research ethics committee.

### Statistical Analysis

Categorical variables such as gender, related ASD comorbid symptoms, were described using frequency and percentage. Continuous variables such as age and daily CBD dose were evaluated for normal distribution using histograms and Q–Q plots. Normally distributed continuous variables were described as mean and standard deviation and skewed variables were expressed as median and interquartile range or range. Length of follow-up was described using a reverse censoring method. A comparison of improvement in symptoms between CBD treatment and conventional treatment was analyzed using binomial test. All statistical analyses were performed using SPSS (IBM Corp 2016. IBM SPSS Statistics for Windows, Version 24.0, Armonk, NY: IBM Corp.).

## Results

Fifty- three patients were included in the study, 45 males (85%) and 8 females (15%). The median age was 11 (range: 4–22) years (Table 1). Median duration of follow-up was 66 (range: 30–588) days. THC median interquartile range (IQR) daily dose was 7 (4–11) mg and CBD median (IQR) daily dose was 90 (45–143) mg.

**Table 1:** Patients characteristics and baseline symptoms.

Characteristics		
Sex, n (%)	Male	45 (84.9)
	Female	8 (15.1)
Age (years), median (range)		11 (4–22)
Medications, n (%)	Stimulants	5 (9.4)
	Typical antipsychotics	6 (11.3)
	Atypical antipsychotics	31 (58.4)
	Anti-epileptic	8 (15)
	Melatonin	4 (7.5)
	Anti-depressant	2 (3.7)
	Other anti-muscarinic	3 (5.6)
	Alpha agonist	1 (1.8)
Days of treatment (days)	Minimum	31
	Maximum	588
	Median	66
Hyperactivity symptoms, n (%)		47 (88.7)
Sleep problems, n (%)		29 (54.7)
Self-injury, n (%)		47 (88.7)
Social communication and reciprocity, n (%)		22 (41.5)
Anxiety, n (%)		26 (49.1)


Six children were excluded because they were treated for less than a month. None of them has discontinued treatment nor had adverse effects. A total of 266 interviews were performed (median 5 interviews per patient).

### After Cannabidiol Administration, Parents Reported on the Various ASD Comorbid Symptoms as Follows

#### Hyperactivity Symptoms

Reports on 38 children with hyperactivity symptoms were recorded. Of them, 68.4% had improvement of symptoms, 28.9% had no change and worsening of symptoms was reported in 2.6%. The improvement was not statistically different from that of the conventional treatment published in the literature (*p* = 0.125).

#### Self-Injury

Of 34 reports on self-injury and rage attacks, 67.6% were reported to experience improvement of symptoms, 23.5% had no change, and worsening of symptoms was reported in 8.8%. There was a borderline significance in improvement of symptoms comparing to the conventional treatment (*p* = 0.063), and no statistical difference in worsening of symptoms (*p* = 0.307).

#### Sleep Problems

Reports on 21 patients with sleep problems were recorded. Of 21 reports, 71.4% improved, 23.8% had no change, and worsening of symptoms was reported in one patient (4.7%). There was no statistically difference comparing to the conventional treatment (*p* = 0.4).

#### Anxiety

Reports on 17 patients with anxiety symptoms were available. Of 17 reports, eight patients (47.1%) had improvement of symptoms, five patients (29.4%) had no change, and worsening of symptoms was reported in four patients (23.5%). There was no statistically difference comparing to the conventional treatment as published in the literature (*p* = 0.232).

#### Overall Improvement

We examined the overall change in ASD comorbidities symptoms of 51 out of 53 patients (Table 2). An overall improvement was reported in 74.5%. No change was reported in 21.6% and worsening in 3.9%. Two patients did not have a report on their overall improvement.

**Table 2:** Overall change in ASD comorbidity symptoms.

Change in symptoms	Frequency
No change, n (%)	11 (21.6)
Improvement, n (%)	38 (74.5)
Worsening, n (%)	2 (3.9)
Total	51
Missing reports	2


#### Adverse Events Reported by the Parents

The most frequent adverse effects were somnolence (*n* = 12) and decreased appetite (*n* = 6) (Table 3).

**Table 3:** Adverse events possibly related to the study, according parent’s reports.

Adverse events	Number of reports
Somnolence	12
Appetite decrease	6
Appetite increase	4
Insomnia	2
Sense abnormality response (to temperature)	2
Eyes blinking	2
Diarrhea	2
Hair loss	1
Nausea	1
Confusion	1
Acne	1
Palpitations	1
Urinary incontinence	1
Eye redness	1
Constipation	1


Five families discontinued follow-up at different time points. Two families reported ineffectiveness and chose to stop treatment; two families decided to continue treatment with a different medical cannabis supplier and in one family the license expired.

## Discussion

In this study, based on recorded data reported by parents of children with ASD, in all four ASD comorbidity symptoms described, parents have reported an overall improvement.

This is one of the first publications on the use of cannabidiol to treat comorbid symptoms of patients with ASD. There are studies which are being conducted these days in several countries such as the United States and Israel, to examine the efficacy and safety of cannabidiol in this population; however, these studies are still ongoing.

The incidence of hyperactivity symptoms in the ASD population ranges between 41 and 78% (Sturm et al., 2004; Murray, 2010). In our study there was an overall improvement of 68.4% [95%CI (51.4–82.5%)] in hyperactivity symptoms as reported by the parents. Conventional treatments for hyperactivity include treatment with methylphenidate. In one study, methylphenidate improved symptoms in 80% (Handen et al., 2000). Comparing the overall improvement in hyperactivity symptoms in children treated with cannabidiol to that achieved with methylphenidate, non-inferiority of cannabidiol was observed (*p* = 0.125).

Self-injurious behavior is common in ASD, with incidence ranging between 35 and 60% (Richards et al., 2016). Our study presented an overall improvement of 67.6% [95%CI (49.5–82.6%)] and worsening of 4.9% [95%CI (1.9–23.7%)] in these symptoms. Currently, atypical antipsychotics are recommended for the treatment serious behavioral symptoms and self-injury (Marcus et al., 2009). Aripiprazole improves symptoms in 82% (any improvement) while 4% presented worsening in symptoms (Marcus et al., 2009). Comparing the overall improvement and worsening in self-injury symptoms in children treated with cannabidiol in our study to that described in the literature with aripiprazole, non-inferiority of cannabidiol was observed (*p* = 0.063, *p* = 0.307, respectively).

Sleep problems in children and adolescents with ASD range between 40 and 80% (Devnani and Hegde, 2015). Conventional treatment with melatonin improved sleep problems in 60% of the patients (Devnani and Hegde, 2015). In our present study cannabidiol was reported to be effective in 71.4% [95%CI (47.8–88.7%)] of the patients in improving sleep problems. Comparing the overall improvement in sleep problems in children treated with cannabidiol to that reported in children treated with melatonin, non-inferiority of cannabidiol was observed (*p* = 0.40).

Anxiety symptoms in children with ASD are common (Sukhodolsky et al., 2008) and are usually controlled with selective serotonin reuptake Inhibitors (SSRI’s) treatment in 55–73% (Moore et al., 2004). In our study, reports on 17 patients with these symptoms were recorded and in 47.1% [95%CI (23.0–72.2%)] of the children an improvement of symptoms was reported. It has been suggested that by improving sleep and disruptive behavior, the motivation and the ability to communicate with the family and the caregivers is improved. Comparing the overall improvement in anxiety symptoms in children treated with cannabidiol to that reported in children treated with SSRI’s, non-inferiority of cannabidiol was observed (*p* = 0.232).

Δ9-THC and CBD are substrates and inhibitors of cytochrome P450 enzymatic pathways relevant to the biotransformation of commonly prescribed psychotropic agents (Rong et al., 2018). Δ9-THC is rapidly metabolized by CYP2C9 and CYP3A4 isoenzymes and CBD is metabolized by CYP2C19 and CYP3A4 (Stout and Cimino, 2014). Data suggest minimal induction of CYPs 1A2, 2C9, 2C19, and 3A4 by Δ9-THC and CBD. However, drug–drug interaction should be considered; phenytoin plasma concentration might be increased, even up to toxic range (Rong et al., 2018). Animal studies have demonstrated that the exposure to Δ9-THC may reverse the neurobehavioral effects of risperidone, which may be less effective (Brzozowska et al., 2017). Other potential drug–drug interactions of cannabidiol include SSRI’s, tricyclic antidepressant and CNS depressants which may result in toxic levels of these medications (Lindsey et al., 2012). In our study, signs and symptoms of toxicity of these medications were not reported.

Most frequent adverse effects, as reported by the parents, were somnolence and change in appetite (Table 3). These symptoms were perceived by the parents as related to the treatment with cannabidiol. All adverse effects were reported to be transient and resolved spontaneously. Several studies have demonstrated that the most common adverse effects associated with CBD use in children and adults are somnolence, change in appetite, diarrhea, and weight changes (Devinsky et al., 2016). Case-studies indicate that cannabinoids may induce acute psychosis which is self-limited over time (Shah et al., 2017); however, cannabis is not considered as the only cause for persistent psychotic disorder. More likely it is the interaction of several factors, such as age at onset of cannabis use, childhood abuse, genetic vulnerability and psychiatric comorbidities which result in psychosis (Wilkinson et al., 2014). Patients with a history of psychotic attacks are more likely to develop cannabis induced psychotic attacks and this should be a contraindication for treatment with CBD (Degenhardt et al., 2018).

Our study has several limitations. All information was based on parents’ reports, with no control group, and there was no objective assessment tool for symptoms changes. We did not have information on the history of ASD symptoms in each patient.

Parents may subjectively report an improvement due to high expectations from the treatment. However, we believe that the main caregivers are the best source to evaluate the child’s status and adverse events. In this population of children with ASD, adverse events are reported by the caregivers rather than the medical staff. Several studies, examining the efficacy and safety of cannabidiol in children with epilepsy, based upon parents’ report, were published in the medical literature (Porter and Jacobson, 2013). Furthermore, our study was conducted on a cohort of patients who were followed up consistently, and not a case series; hence, the rates of treatment success or failure are calculated based on a genuine denominator.

## Conclusion

Children with ASD commonly have comorbid symptoms such as aggression, hyperactivity and anxiety. There is an increase in the use of cannabidiol in children with ASD. Based on parents’ reports, our findings suggest that cannabidiol may be effective in improving ASD comorbid symptoms; However, CBD efficacy and safety should be further evaluated in children with ASD in large-scale clinical trials.

## Author Contributions

DB, OS, TD-H, and MB performed the major research in equal contribution. TZ-B provided the statistical analysis. DF, GK, and NS contributed as consultants.

## Conflict of Interest Statement

The authors declare that the research was conducted in the absence of any commercial or financial relationships that could be construed as a potential conflict of interest.
